# Neurocognitive outcomes of children exposed to and living with HIV aged 3–5 years in Kilifi, Kenya

**DOI:** 10.3389/frph.2023.1193183

**Published:** 2023-09-05

**Authors:** Esther Jebor Chongwo, Catherine J. Wedderburn, Moses Kachama Nyongesa, Antipa Sigilai, Paul Mwangi, Janet Thoya, Rachel Odhiambo, Katana Ngombo, Beatrice Kabunda, Charles R. Newton, Amina Abubakar

**Affiliations:** ^1^ Institute for Human Development, Aga Khan University, Nairobi, Kenya; ^2^ Department of Clinical Research, London School of Hygiene and Tropical Medicine, London, United Kingdom; ^3^ Department of Paediatrics and Child Health and Neuroscience Institute, University of Cape Town, Cape Town, South Africa; ^4^ KEMRI-Wellcome Trust Research Programme, Centre for Geographic Medicine Research (Coast), Kilifi, Kenya; ^5^ Department of Psychiatry, University of Oxford, Oxford, United Kingdom

**Keywords:** HIV, children, caregivers, mental health, nutrition, parenting, neurocognitive

## Abstract

**Introduction:**

Globally, 1.7 million children are living with HIV, with the majority of them residing in sub-Saharan Africa. Due to reduced rates of vertical transmission of HIV, there is an increasing population of children born to HIV-infected mothers who remain uninfected. There is a growing concern around the development of these children in the antiretroviral therapy era. This study examined the neurocognitive outcomes of children who are HIV-exposed infected (CHEI), HIV-exposed uninfected (CHEU) and HIV-unexposed uninfected (CHUU) and explored the relationship between child neurocognitive outcomes and child's biomedical and caregivers’ psychosocial factors.

**Methods:**

CHEI, CHUU and CHEU aged 3–5 years and their caregivers were recruited into the study. Neurocognitive outcomes were assessed using a validated battery of assessments. One-way analysis of variance and covariance (ANOVA and ANCOVA) were used to evaluate differences among the three groups by neurocognitive outcomes. Linear regression models were used to investigate the association between child neurocognitive outcomes and biomedical factors (nutritional status, HIV disease staging) and caregivers’ psychosocial factors [symptoms of common mental disorders (CMDs) and parenting behaviour].

**Results:**

The study included 153 children and their caregivers: 43 (28.1%) CHEI, 52 (34.0%) CHEU and 58 (39.9%) CHUU. ANOVA and ANCOVA revealed a significant difference in cognitive ability mean scores across the child groups. *Post hoc* analysis indicated that CHEU children had higher cognitive ability mean scores than the CHUU group. Better nutritional status was significantly associated with higher cognitive ability scores (*β* = 0.68, 95% CI [0.18–1.18], *p* = 0.008). Higher scores of CMDs were negatively associated with inhibitory control (*β* = −0.28, 95% CI [−0.53 to 0.02], *p* = 0.036). While comparing HIV stages 2 and 3, large effect sizes were seen in working memory (0.96, CI [0.08–1.80]) and cognitive ability scores (0.83 CI [0.01–1.63]), indicating those in stage 3 had poor performance.

**Conclusions:**

Neurocognitive outcomes were similar across CHEI, CHEU and CHUU, although subtle differences were seen in cognitive ability scores where CHEU had significantly higher cognitive mean scores than the CHUU. Well-designed longitudinal studies are needed to ascertain these findings. Nonetheless, study findings underscore the need for strategies to promote better child nutrition, mental health, and early antiretroviral therapy initiation.

## Introduction

HIV/AIDS remains a major public health issue and an important cause of disability globally (1, 2). Worldwide, approximately 38.4 million people live with HIV, the majority residing in sub-Saharan Africa (SSA) (3–5). Approximately 1.7 million children live with HIV globally (6). Kenya is one of the SSA countries with a high burden of HIV (7) with an average prevalence of 4.8% and an estimated 1.4 million people living with the disease (8). Among those living with HIV, approximately 83,000 are children aged 0–14 years (8).

The roll-out of antiretroviral therapy (ART) has tremendously prolonged the survival of individuals living with HIV, including children (9). With the decline in mortality rates attributable to ART, the impact of HIV on disability is becoming increasingly important. As children who are HIV-exposed and infected (CHEI) on ART survive longer, their neuropsychological functioning has become an area of great interest. HIV has been shown to cross the blood-brain barrier and enter the central nervous system (CNS) causing cerebral encephalopathy and impairment of the cerebral integrity which leads to neurodevelopmental delays (10, 11). This may be further exacerbated by infectious co-morbidities due to immune suppression. Parental HIV can also affect child-rearing practices with major implications for child development.

Neurodevelopmental delays reported in CHEI include impairment in cognition, language and motor functioning, among others (12). Before the availability of ART, more than 50% of CHEI had poor neurocognitive outcomes, in particular, delays in language and motor activity (13). Neurocognitive outcomes amongst CHEI in the modern ART era are less well described. A study by Whitehead and colleagues showed that CHEI infants on ART had greater neurodevelopmental impairment compared to children who were HIV-unexposed and uninfected (CHUU), implying that neurodevelopmental delays persist in the ART era (14). Generally, there has been inconsistent evidence on the neurocognitive outcomes of CHEU and CHUU. Some studies have suggested impairments in motor, language, cognitive and behavioural outcomes in CHEU compared to CHUU (15–17). In contrast, others have not reported any differences between the two groups (18–20). However, with early intervention the adverse effects of HIV can be mitigated. For instance, a study conducted by Strelau and colleagues in South Africa demonstrated that implementing a neurodevelopmental stimulation intervention for CHEI treated early CHEI resulted in a significant reduction in neurodevelopmental delays (7). These findings suggest that early initiation of ART alongside other early childhood stimulation programs has the potential to significantly enhance child developmental outcomes.

In SSA, data on the neurocognitive functioning of CHEI receiving ART in comparison with children who are HIV-exposed uninfected (CHEU) and CHUU pre-schoolers is scarce, yet the region bears a high burden of the disease (21). Data from studies conducted in other settings, particularly those in high-income countries, cannot be directly extrapolated to the SSA context as there exist significant differences in the social support structures, health care systems and the constellation of risks factors for child neurodevelopment including negative environmental factors such as pervasive poverty, malnutrition and malaria infection (22). Although previous studies on child neurodevelopment in the context of HIV have been conducted in Kenya, these have only assessed CHEI without recruiting appropriate comparison groups. One of these studies followed up CHEI for 2 years and observed that a compromised immune system was associated with poor developmental milestones for these children (23). Another study by Gomez and colleagues among newly diagnosed CHEI aged below 5.5 years found significantly improved neurodevelopment on the commencement of ART (24).

Despite HIV infection, improved nutrition has been linked with better cognitive development in CHEI infants (23–25), but there is a lack of comparative data on the nutritional status between children living with HIV and other child groups such as CHEU and CHUU from Kenya. Research on the unique neurodevelopmental profile of children living with HIV compared to their uninfected peers in low- and middle-income countries is needed since early child development (before the age of 5 years) is known to be critical for future scholastic achievement, social skills, behavioural outcomes, and quality of life (26–28). In addition, this stage is a timely period to intervene and take opportunity of the window of plasticity (29).

Furthermore, with strategies such as the prevention of mother-to-child transmission of HIV (PMTCT), there is a growing population of children born to HIV-infected mothers who remain uninfected due to the protective effects of ART in pregnancy. There are currently an estimated 15.9 million CHEU worldwide (30). Growing evidence suggests that CHEU have higher mortality and morbidity compared to CHUU (31). While the long-term neurocognitive outcomes of this population are unclear, there are reports of adverse outcomes in the early years, particularly in language and motor domains (32–35). These observations could plausibly be explained by: foetal exposure to HIV virus or maternal immune activation *in utero*; ART exposure *in utero* and prophylaxis during the neonatal period; or living in an HIV-affected household compounded with adverse psychosocial, socioeconomic and environmental factors (29, 32).

This study aimed to examine the neurocognitive outcomes of children aged 3–5 years exposed to and living with HIV compared to a representative sample of HIV-unexposed uninfected children on the Kenyan coast. The study also examined the relationship between child neurocognitive outcomes and the child's biomedical factors (nutritional status, HIV disease staging) and caregivers’ psychosocial factors [symptoms of common mental disorders (CMDs) and parenting behaviour].

## Methods

### Study site

The study was conducted in Kilifi County, Kenya, at the Comprehensive Care and Research Clinic (CCRC), Kilifi County Hospital, through the Centre for Geographic Medicine Research-Coast (CGMR-C). In Kilifi County, a largely rural setting, the overall prevalence of HIV is estimated as 4.5% (higher in women than men, 6.4% vs. 2.7%) and approximately 3,500 children live with the disease (36). Children and caregivers attending the CCRC were targeted for recruitment. The CCRC provides comprehensive care for adults and children living with or exposed to HIV. Some of the services offered include initiation and refill of ART medication, ART adherence monitoring, treatment and management of opportunistic infections, family planning, cervical cancer screening, nutritional counselling as well as HIV testing and counselling. Within the CGMR-C, is a neuro-assessment unit started in 1992 with well-trained and experienced staff for administering measures of neurological, mental, and cognitive functioning.

### Sample and sampling procedures

Power calculations for this study were conducted based on data from a previous study by Fishkin et al. to identify the effects of HIV on executive function (EF) (37). Using an effect size of 0.50 and a significance level of 0.05, the present study of 153 children (and caregivers) including 43 CHEI, 52 CHEU and 58 HIV-unexposed controls had a power of 85% to detect group differences.

The inclusion criteria for the children included in this study were: (i) age range 36–60 months; (ii) confirmation of positive HIV status for the CHEI and confirmation of negative HIV status for CHEU from the health records (iii) their caregivers spoke either Kiswahili, English or Mijikenda (local vernacular); and (iv) gave informed consent for child participation. Random sampling was used to recruit CHEI (*n* = 43) and CHEU (*n* = 52) during their scheduled HIV clinic visits at the Kilifi County Referral Hospital (CCRC). All CHEI had positive HIV tests confirmed at 18 months using the standard HIV antibody tests and virology assays. The CHEU group were those born to mothers living with HIV, but children tested HIV negative at 18 months. These tests are part of the routine clinical care under the PMTCT package. This information was confirmed using the documentation in the antenatal clinic (ANC) cards. Caregivers who consented for their children to take part in the study were recruited and invited to the neuro-assessment unit for assessments.

The CHUU group (*n* = 58) consisted of children not infected with or exposed to HIV. They were selected randomly from an existing database, the Kilifi Health and Demographic Surveillance System (KHDSS) (38). To ensure comparability, these children were matched to the CHEI based on age and geographical location. Subsequently, a field worker approached their caregivers for consenting at home. Due to ethical justifications and logistical difficulties, the HIV status of community controls was not directly tested during the time of assessment, but caution was exercised to minimise the chances of recruiting HIV-positive children. First, only children whose mothers had negative HIV tests during ANC visits (confirmed through clinic cards) were recruited. Second, a detailed history was taken to exclude children with severe childhood illnesses. Also, taking into account the 9% prevalence rate of HIV infection in mothers (39), high child mortality rate of CHEI (up to 50% by their second birthday) and expected mother-to-child transmission of 25%–40% (25), it was estimated that a maximum of two children would likely be HIV-positive in the control group. This number was deemed unlikely to invalidate the study results.

### Study procedures

An overview of the study was introduced to all patients seeking services at the CRCC during the routine morning health talks (given before service initiation). Caregivers of CHEI and CHEU aged 3–5 years who showed interest in the study were approached by a member of the study team and taken through the study information sheet in detail in either the national (Swahili) or local (Giriama) language. Upon acceptance of study participation and signing of informed consent, the participants were booked for assessment dates at the neuro assessment clinic where the assessments were done. On the day of assessment, reaffirmation of consent was done before the start of any study-related activity. Study questionnaires and neurocognitive assessments were administered by a team of trained and experienced research assistants.

### Ethical approval

Permission to conduct this study in Kenya was sought and granted by the Institutional Review Board of the Kenya Medical Research Institute (KEMRI) called the Scientific and Ethics Review Unit (SERU), reference number: SSC No.2210. Information about the study was fully explained to all participants (in this case caregivers) and provided written informed consent for participation in their local languages (Swahili and Giriama).

### Measures

#### Child and maternal socio-demographics

Data on the child's age, sex, and schooling were collected. Information on maternal schooling and household socio-economic status was also documented.

#### Child clinical data

All children's weight and height were taken using SECA digital scale. A medical examination of the children was done before the interview. This included physical examination of the participants including ear exams. Additionally, a review of previous medical history was done for all the children using a standard clinical questionnaire which had questions including history and the number of previous hospital admissions.

#### HIV disease staging

The World Health Organization's (WHO) clinical staging of HIV was used to assess the disease progression in CHEI. HIV is categorized from stage 1 (later/early stage) to stage 4 (advanced stage of AIDS) based on clinical signs and symptoms (40). This information was extracted from the medical records of the participants at the CCRC, with the prior permission of their caregivers.

#### Height for age (HAZ)

The height for age *Z*-scores were computed using Anthro software following WHO standards [24]. HAZ was selected in this study because it measures stunting which has been found to be a good indicator of chronic malnutrition. In this study, a cut-off HAZ ≤2 SD was used. A systematic review by Perkins and colleagues demonstrated evidence of an association between stunting and child neurodevelopment (41).

### Child neurocognitive outcome measures

The following battery of neurocognitive assessment tests was administered.

#### A-not-B task

This is a simple measure of cognitive flexibility where the child identifies the hidden place for an object. The object is hidden in the child's view in one of two sites and the choice is then re-presented after a short delay to the child. The object's hiding place is changed between trials. The task is intended to elicit the A-not-B error, whereby the child continues to select a previously successful response despite seeing a new hiding place. The tool has 10 items, each scoring “one” for a correct response and “zero” for an incorrect response. The A-not-B task has been well-validated as a measure of cognitive flexibility, linked to frontal lobe functioning (42–44). In this study, the recommended procedures described by Espy and colleagues (45) were followed.

#### Wisconsin card sorting test

This task assesses set-shifting (46). The children were instructed to sort cards by numbers or by flowers and were scored based on whether they got them correct. The tool has 32 items each scoring “one” for a correct response and “zero” for an incorrect response.

#### Number recall test

This is a measure of working memory in which participants are presented with numbers and after a period of delay, they are asked to remember the numbers as many as possible (47). If they successfully repeat, the order list becomes longer. The tool has 24 items, each scoring “one” for a correct response and “zero” for an incorrect response. The tool has been validated in Kenyan children and showed a test-retest reliability of 0.70 (48).

#### Big-small stroop test

This task uses a set of pictures showing big or small circles in black and the child is required to say the size of the picture as instructed by the assessor. This test was used because it is fairly easily understood by children and also, the picture sizes are distinctively opposite (49). The tool has 24 items each scoring “one” for a correct response.

#### Block design task

Adapted from the third edition of the British Ability Scales (50), this tool measures cognitive ability. A child is required to construct wooden design blocks as shown by the assessor. The task has 16 items, and a correctly constructed design is awarded a point, giving a maximum score of 16. The tool has been validated in Ugandan children and demonstrated high reliability of 0.82 (51).

#### Picture vocabulary test

This tool has 24 items each with four pictures drawn in black and white: a target picture, a phonological distracter, a visual or semantic distracter, and an unrelated distracter (52). A child points to one of the items on every page as instructed by the assessor. This tool was adapted and validated in Kilifi, Kenya as a measure of receptive language and demonstrated good internal consistency reliability of 0.86 (48).

For these neurocognitive tests, all the assessments were conducted in Kiswahili or Giriama, based on what was most convenient to the participant. The number recall test was strictly carried out in Kiswahili to avoid method bias.

### Caregiver measures

#### Shona symptoms questionnaire (SSQ)

This is a 14-item screening tool for symptoms of common mental disorders (CMDs) that was developed in Zimbabwe (53). The measure has been used in various African settings (54–56) including Kenya (57). It is based on a “yes/no” response option, asking about symptoms such as suicidal ideations, failure to concentrate, unhappiness and thinking too much, among others, over the past 1 week. Higher scores indicate poor mental health. The Cronbach's alpha for SSQ in this study was 0.83.

Parenting behaviour was measured using 7 items (based on a “yes/no” response option) adapted from the UNICEF childcare module of the multiple indicator survey (58). We used items aimed at evaluating the cognitive stimulation that the child receives from the caregiver, for example, involvement with the child in activities such as reading, storytelling and singing. A score of one is given for a “No” response and two for a “Yes” response. Higher scores indicate better parenting behaviour.

### Statistical analysis

Data analysis was carried out using STATA (version 15). Percentages and frequencies were used to summarize socio-demographic data. One-way analysis of variance (ANOVA) was utilized to identify group differences for continuous variables. Pearson's chi-squared test was used to assess group differences for categorical variables. ANOVA and analysis of covariance (ANCOVA), adjusting for the child's age and schooling, were done to identify group differences in the children's neurocognitive outcomes. Further, *post hoc* analysis using Bonferroni pairwise comparison was done for the scores that showed significant group differences in the ANOVA analysis. Linear regression models were used to investigate the association between child neurocognitive (outcome) and the following exposures: (i) the child's biomedical factors (nutritional status and HIV disease staging); and (ii) caregivers’ psychosocial factors (symptoms of CMDs and parenting behaviour). All the predictors were included in the adjusted model based on previous literature findings. We further computed Cohen's *d* to compare the effect sizes for child neurocognitive scores by HIV disease staging in the CHEI sub-group.

## Results

### Participant characteristics

Table 1 provides details of the socio-demographic characteristics of the participants. A total of one hundred and fifty-three (153) children and their primary caregivers participated in this study including 43 CHEI children and caregivers, 52 CHEU and 58 CHUU. Table 1 summarizes participant characteristics by HIV infection status. The mean age (SD) of the children was 50.97 (9.70) months. Approximately half of the children were girls (52.3%) and the majority attended school (63.6%). Of the caregivers, 45.1% had a secondary or higher level of education. There was a statistically significant difference in the mean CMDs scores across the three caregiver groups (CHEI, CHEU and CHUU), showing higher CMD scores in caregivers of CHEI and CHEU groups. The mean scores of positive parenting behaviour also significantly differed across the three caregiver groups (CHEI, CHEU and CHUU).

**Table 1:** Socio-demographic characteristics of the children and caregivers, by HIV infection status.

	Whole sample	CHUU	CHEU	CHEI	*p*-value
	*N* = 153	*N* = 58	*N* = 52	*N* = 43	
Child characteristics					
Age in months *M* (SD)	50.97 (9.70)	52.03 (7.51)	49.02 (10.85)	51.91 (10.68)	0.203
Gender *n* (%)					
Male	73 (47.71)	30 (51.72)	24 (46.15)	19 (44.19)	0.726
Female	80 (52.29)	28 (48.28)	28 (53.85)	24 (55.81)	** **
Child schooling *n* (%)					
No	55 (36.42)	24 (41.38)	16 (31.37)	15 (35.71)	0.553
Yes	96 (63.58)	34 (58.62)	35 (68.63)	27 (64.29)	** **
Nutritional status *M* (SD)					
Height for age	−1.52 (1.34)	−1.57 (1.08)	−1.32 (1.63)	−1.69 (1.31)	0.419
HIV disease stage					
Stage 1	NA	NA	NA	11 (25.00)	
Stage 2	NA	NA	NA	22 (50.00)	
Stage 3	NA	NA	NA	10 (25.00)	
Stage 4	NA	NA	NA	0 (0.00)	
Caregiver characteristics respondent *n* (%)					
Mother	132 (86.84)	49 (84.48)	46 (90.20)	37 (86.05)	0.667
Others	20 (13.16)	9 (15.52)	5 (9.80)	6 (13.95)	
CMD scores *mean (SD)*	19.04 (3.60)	17.28 (2.74)	19.90 (3.4)	20.37 (4.00)	**<0** **.** **001**
Parenting behaviour scores *mean (SD)*	11.14 (1.84)	11.60 (1.70)	11.03 (1.68)	10.67 (2.09)	**0** **.** **037**
Caregiver level of education					
None	21 (13.82)	10 (17.54)	7 (13.46)	4 (9.30)	0.867
Primary	62 (40.79)	25 (43.86)	20 (38.46)	17 (39.53)	
Secondary	27 (17.76)	8 (14.04)	10 (19.23)	9 (20.93)	
Post-secondary	42 (27.63)	14 (24.56)	15 (28.85)	13 (30.23)	

CHUU, HIV unexposed uninfected; CHEU, HIV-exposed uninfected; HEI, HIV-exposed infected (HIV status for the children); CMD, common mental disorders; NA, not applicable.

Bold values represent *p*-values <0.05.

### Neurocognitive profiles of CHEI, CHEU and CHUU

Table 2 presents the mean (SD) scores for the neurocognitive battery tests. ANOVA results showed no statistically significant differences in the scores of receptive language, inhibitory control, set-shifting and cognitive flexibility across the three participant groups. However, there was a significant group difference in cognitive ability scores, *p* = 0.002. *Post hoc* analysis showed significantly higher cognitive ability scores among CHEU compared to (i) CHUU (*p* < 0.03801), and (ii) CHEI (*p* = 0.0028). After controlling for the child's characteristics (age, sex and schooling) and caregiver's characteristics (education level, CMD scores and parenting behaviour), ANCOVA results revealed that cognitive ability scores differed significantly between HIV groups, *p* = 0.006 (Table 2).

**Table 2:** Neurocognitive scores across the child groups.

Measure	CHUU	CHEU	CHEI	*p*-value (unadjusted^a^)	*p*-value (adjusted^b^)
	Mean (SD)	Mean (SD)	Mean (SD)		
	Median (IQR)	Median (IQR)	Median (IQR)		
Receptive language	15.79 (3.43)	14.57 (4.57)	14.73 (4.80)	0.293	0.585
Cognitive ability	7.05 (3.65)	9.73 (3.96)	7.58 (3.94)	**0** **.** **002**	**0** **.** **006**
Inhibitory control	10.17 (5.03)	11.27 (5.23)	11.19 (5.29)	0.509	0.115
Working memory	6.56 (2.67)	6.59 (3.14)	6.19 (3.04)	0.812	0.240
Set shifting	10.98 (6.37)	10.98 (5.18)	11.50 (6.41)	0.915	0.992
Cognitive flexibility	8.85 (1.00) 9 (6–10)	8.73 (1.25) 9 (6–10)	8.86 (1.29) 9 (4–10)	0.832	0.791

Adjusted for the child's characteristics (age, sex, and schooling) and caregiver's characteristics (education level, CMD scores and parenting behaviour). Bolded *p*-value <0.05.

^a^ ANOVA.

^b^ ANCOVA.

### Relationship between child neurocognitive outcomes with child biomedical and caregiver's psychosocial factors

#### Unadjusted analyses

Table 3 presents the results of the unadjusted linear regression. The results revealed a significant association between the child's neurocognitive outcomes and HIV group, child age, child schooling, sex, nutrition status, HIV disease staging, caregiver level of education and positive parenting behaviour. The cognitive ability score for the CHEU group was 2.76 units higher than the CHUU group (*β* = 2.76, 95% CI [1.14–4.20], *p* = 0.001). Every 1-month increase in child's age was significantly associated with increased receptive language (*β* = 0.17, 95% CI [0.10–0.24], *p* < 0.001), working memory (*β* = 0.14, 95% CI [0.10–0.19], *p* < 0.001), inhibitory control (*β* = 0.17, 95% CI [0.07–0.26], *p* = 0.001), cognitive ability (*β* = 0.07, 95% CI [0.00–0.14], *p* = 0.049), set-shifting (*β* = 0.24, 95% CI [0.14–0.35], *p* < 0.001), and cognitive flexibility (*β *= 0.04, 95% CI [0.02–0.06], *p* < 0.001) scores, respectively. There was a significant increase in the receptive language (*β *= 0.75, 95% CI [0.23–1.27], *p* = 0.005), working memory (*β* = 0.41, 95% CI [0.02–0.79], *p* = 0.040), inhibitory control (*β* = 1.07, 95% CI [0.40–1.75], *p* = 0.002), cognitive ability (*β* = 0.91, 95% CI [0.42–1.41], *p* < 0.001), and set-shifting (*β* = 1.02, 95% CI [0.18–1.86], *p* = 0.018) scores for every unit increase in nutritional status. Children who were attending school had significantly increased scores in receptive language (*β* = 4.08, 95% CI [2.77–5.39], *p* < 0.001), working memory (*β* = 2.60, 95% CI [1.59–3.60], *p* < 0.001), inhibitory control (*β* = 4.89, 95% CI [3.18–6.60], *p* < 0.001), cognitive ability (*β* = 3.37, 95% CI [2.06–4.68], *p* < 0.001), set-shifting (*β* = 5.92, 95% CI [3.80–8.05], *p* < 0.001), and cognitive flexibility (*β* = 0.68, 95% CI [0.23–1.04], *p* = 0.002) compared to those who did not attend school. There was a significant increase in child receptive language (*β* = 2.51, 95% CI [0.21–4.81], *p* = 0.033) and inhibitory control scores (*β* = 3.54, 95% CI [0.67–6.40], *p* = 0.016) for caregivers with post-secondary education compared to those with no education. A unit increase in positive parenting behaviour significantly increased child's receptive language (*β* = 0.67, 95% CI [0.30–1.03], *p* < 0.001), inhibitory control (*β* = 0.68, 95% CI [0.22–1.15], *p* = 0.004) and working memory (*β* = 0.46, 95% CI [0.19–0.73], *p* = 0.001) scores.

**Table 3:** Unadjusted linear regression for the association between neurocognitive outcomes and child biomedical factors and caregiver's psychosocial factors.

	Receptive language		Working memory		Inhibitory control		Cognitive ability		Set-shifting		Cognitive flexibility	
	*β* (95% CI)	*p*-Value	*β* (95% CI)	*p*-Value	*β* (95% CI)	*p*-Value	*β* (95% CI)	*p*-Value	*β* (95% CI)	*p*-Value	*β* (95% CI)	*p*-value
Child characteristics												
HIV status												
CHUU (reference)	*Reference*											
CHEI	−0.06 (−2.82 to 0.71)	0.238	−0.37 (−1.68 to 0.94)	0.576	1.02 (−1.26 to 3.30)	0.378	0.52 (−1.07 to 2.12)	0.518	0.52 (−2.18 to 3.22)	0.703	0.01 (−0.49 to 0.50)	0.979
CHEU	−1.22 (−2.89 to 0.46)	0.153	0.03 (−1.17 to 1.23)	0.959	1.11 (−0.97 to 3.18)	0.294	2.76 (1.14–4.20)	**0** **.** **001**	−0.01 (−2.53 to 2.52)	0.997	−0.13 (−0.59 to 0.34)	0.591
Child age	0.17 (0.10–0.24)	**<0** **.** **001**	0.14 (0.10–0.19)	**<0** **.** **001**	0.17 (0.07–0.26)	**0** **.** **001**	0.07 (0.00–0.14)	**0** **.** **049**	0.24 (0.14–0.35)	**<0** **.** **001**	0.004 (0.02–0.06)	**<0** **.** **001**
Child sex												
Male	*Reference*	** **		** **		** **		** **		** **		** **
Female	0.12 (−1.30–1.56)	0.861	0.89 (−0.13 to 1.91)	0.086	0.47 (−1.33 to 2.26)	0.609	−0.10 (−1.44 to 1.26)	0.895	0.75 (−1.40 to 2.90)	0.490	0.62 (0.23–1.00)	**0** **.** **002**
Child schooling												
No	*Reference*											** **
Yes	4.08 (2.77–5.39)	**<0** **.** **001**	2.60 (1.59–3.60)	**<0** **.** **001**	**4.89** **(3.18–6.60)**	**<0** **.** **001**	**3.37** **(2.06–4.68)**	**<0** **.** **001**	**0.63** **(0.23–1.04)**	**0** **.** **002**	0.63 (0.23–1.04)	**0** **.** **002**
Nutrition (height for age)	0.75 (0.23–1.27)	**0** **.** **005**	0.41 (0.02–0.79)	**0** **.** **040**	1.07 (0.40–1.75)	**0** **.** **002**	0.91 (0.42–1.41)	**<0** **.** **001**	1.02 (0.18–1.86)	**0** **.** **018**	0.03 (−0.12 to 0.18)	0.729
Caregiver characteristics												
Education level												
None	*Reference*			** **		** **		** **		** **		
Primary	1.42 (−0.76 to 3.60)	0.201	0.74 (−0.84 to 2.32)	0.358	1.84 (−0.88 to 4.57)	0.183	1.15 (−0.90 to 3.22)	0.269	1.30 (−2.10 to 4.71)	0.451	0.23 (−.38 to 0.84)	0.453
Secondary	1.36 (−1.13 to 3.85)	0.283	−0.14 (−1.94 to 1.67)	0.880	1.80 (−1.30 to 4.91)	0.252	0.66 (−1.70 to 3.02)	0.580	2.43 (−1.45 to 6.32)	0.218	0.03 (−0.66 to 0.73)	0.932
Post-secondary	**2.51** **(0.21–4.81)**	**0** **.** **033**	1.40 (−0.27 to 3.06)	0.100	**3.54** **(0.67–6.40)**	**0** **.** **016**	2.15 (−0.02 to 4.32)	0.052	3.22 (−0.36 to 6.81)	0.078	0.22 (−0.42 to 0.86)	0.500
CMDs	−0.17 (−0.37 to 0.04)	0.106	−0.03 (−0.18 to 0.12)	0.698	−0.13 (−0.39 to 0.13)	0.323	0.08 (−0.11 to 0.28)	0.400	−0.10 (−0.40 to 0.21)	0.536	−0.02 (−0.08 to 0.04)	0.467
Parenting behavior	0.67 (0.30–1.03)	**<0** **.** **001**	0.46 (0.19–0.73)	**0** **.** **001**	0.68 (0.22–1.15)	**0** **.** **004**	0.31 (−0.04 to 0.66)	0.087	0.57 (−0.01 to 1.14)	0.052	0.09 (−0.02 to 0.19)	0.109
HIV disease stage												
Stage 1	*Reference*											** **
Stage 2	1.78 (−2.06 to 5.62)	0.352	0.29 (−2.40 to 2.97)	0.829	−1.41 (−6.14 to 3.32)	0.547	0.49 (−1.54 to 4.52)	0.324	−1.63 (−7.30 to 4.05)	0.563	0.66 (−0.33 to 1.66)	0.183
Stage 3	−0.59 (−4.77 to 3.79)	0.817	−2.38 (−5.37 to 0.61)	0.114	−3.65 (−8.90 to 1.59)	0.165	−1.37 (−4.79 to 2.05)	0.421	−4.85 (−11.15 to 1.45)	0.126	1.17 (0.03–2.31)	**0** **.** **044**

Bold values represents *p*-value <0.05. CHUU, HIV unexposed uninfected; CHEU, HIV-exposed uninfected; HEI, HIV-exposed infected; CMDs, common mental disorders. The HIV disease stage is only for those who are HIV positive.

#### Adjusted analyses

Table 4 shows the results for the adjusted linear regression model. In the multiple linear models, results showed that the child's HIV status, age and nutritional status were significantly associated with child neurocognitive outcomes. The CHEU group had a higher score in inhibitory control (*β *= 2.16, 95% CI [0.09–4.23], *p *= 0.041) and cognitive ability (*β *= 2.55, 95% CI [0.92–4.18], *p *= 0.002) compared to the CHUU group. For every 1-month increase in the child's age, there was a significant increase in the receptive language (*β *= 0.11, 95% CI [0.04–0.18], *p *= 0.004), working memory (*β *= 0.12, 95% CI [0.07–0.17], *p* < 0.001), inhibitory control (*β *= 0.12, 95% CI [0.02–0.21], *p *= 0.017), set shifting (*β *= 0.19, 95% CI [−0.07 to 0.30], *p *= 0.001), and cognitive flexibility (*β *= 0.03, 95% CI [0.01–0.05], *p *= 0.031) scores. A child being female was significantly associated with increased cognitive flexibility scores (*β *= 0.48, 95% CI [0.09–0.87], *p *= 0.031). Child school attendance was significantly associated with increased receptive language (*β *= 2.82, 95% CI [1.29–4.35], *p* < 0.001), working memory (*β *= 1.14, 95% CI [0.05–2.24], *p *= 0.040), inhibitory control (*β *= 3.27, 95% CI [1.26–5.27], *p *= 0.002), and set-shifting (*β *= 4.20, 95% CI [1.67–6.72], *p *= 0.001) scores. A unit increase in child nutritional status was significantly associated with increased cognitive ability scores (*β *= 0.68, 95% CI [0.18–1.18], *p *= 0.008). Increasing CMDs scores were significantly associated with reduced inhibitory control scores (*β* = −0.28, 95% CI [−0.53 to 0.02], *p *= 0.036).

**Table 4:** Adjusted multivariable regression for the association between neurocognitive outcomes and child biomedical and caregiver's psychosocial factors.

	Receptive language		Working memory		Inhibitory control		Cognitive ability		Set shifting		Cognitive flexibility	
	*β* (95% CI)	*p*-Value	*β* (95% CI)	*p*-Value	*β* (95% CI)	*p*-Value	*β* (95% CI)	*p*-Value	*β* (95% CI)	*p*-Value	*β* (95% CI)	*p*-value
Child characteristics												
HIV status												
CHUU (reference)	*Reference*											
CHEI	−0.88 (−2.60 to 0.84)	0.312	−0.06 (−1.29 to 1.17)	0.576	1.60 (−0.69 to 3.90)	0.170	0.66 (−1.08 to 2.40)	0.453	0.09 (−2.67 to 2.85)	0.951	0.19 (−0.36 to 0.74)	0.496
CHEU	−0.58 (−2.18 to 1.02)	0.474	0.78 (−0.35 to 1.91)	0.172	2.16 (0.09–4.23)	**0** **.** **041**	2.55 (0.92–4.18)	**0** **.** **002**	0.16 (−2.38 to 2.69)	0.902	0.11 (−0.40 to 0.61)	0.678
Child age	0.11 (0.04–0.18)	**0** **.** **004**	0.12 (0.07–0.17)	**<0** **.** **001**	0.12 (0.02–0.21)	**0** **.** **017**	0.04 (−0.04 to 0.11)	0.312	0.19 (0.07–0.30)	**0** **.** **001**	0.03 (0.01–0.05)	**0** **.** **031**
Child sex												
Male	*Reference*	** **		** **		** **		** **		** **		** **
Female	−0.09 (−1.32 to 1.14)	0.882	0.53 (−0.34 to 1.41)	0.228	0.15 (−1.47 to 1.76)	0.856	−0.10 (−1.34 to 1.14)	0.875	0.18 (−1.79 to 2.15)	0.856	0.48 (0.09–0.87)	**0** **.** **017**
Child schooling												
No	*Reference*											** **
Yes	2.82 (1.29–4.35)	**<0** **.** **001**	1.14 (0.05–2.24)	**0** **.** **040**	3.27 (1.26–5.27)	**0** **.** **002**	2.48 (0.94–4.03)	**0** **.** **002**	4.20 (1.67–6.72)	**0** **.** **001**	0.40 (−0.09 to 0.88)	0.108
Nutrition (height for age)	0.47 (−0.03 to 0.96)	0.063	0.28 (−0.07 to 0.63)	0.115	0.45 (−0.22 to 1.13)	0.184	0.68 (0.18–1.18)	**0** **.** **008**	0.64 (−0.18 to 1.47)	0.126	−0.02 (−0.18 to 0.14)	
Caregiver characteristics												
Education level												
None	*Reference*			** **		** **		** **		** **		
Primary	1.77 (−0.10 to 3.63)	0.064	1 (−0.36 to 2.36)	0.147	2.41 (−0.07 to 4.88)	0.057	1.21 (−0.68 to 3.09)	0.208	1.75 (−1.37 to 4.87)	0.269	0.38 (−0.21 to 0.97)	0.201
Secondary	0.96 (−1.23 to 3.16)	0.387	−0.13 (−1.68 to 1.43)	0.873	1.15 (−1.75 to 4.05)	0.434	−0.15 (−2.37 to 2.07)	0.896	2.23 (−1.42 to 5.88)	0.228	0.26 (−0.43 to 0.96)	0.450
Post-secondary	1.95 (−0.09 to 3.99)	0.061	1.02 (−0.46 to 2.51)	0.175	2.76 (0.01–5.52)	0.050	1.15 (−0.91 to 3.21)	0.273	2.73 (−0.72 to 6.18)	0.120	0.28 (−0.37 to 0.92)	0.398
CMDs	−0.14 (−0.33 to 0.06)	0.161	−0.07 (−0.21 to 0.07)	0.326	−0.28 (−0.53 to −0.02)	**0** **.** **036**	−0.03 (−0.23 to 0.16)	0.737	−0.16 (−0.47 to 0.15)	0.302	−0.04 (−0.10 to 0.02)	0.206
Parenting behavior	0.11 (−0.28 to 0.49)	0.587	0.18 (−0.09–0.45)	0.187	0.18 (−.32 to 0.68)	0.480	−0.10 (−0.49 to 0.28)	0.596	−0.11 (−0.73 to 0.50)	0.714	0.03 (−0.09 to 0.16)	0.590

Bold values represent *p*-values <0.05.

Table 5 shows the effect sizes from a comparison of child neurocognitive scores by HIV disease staging. While comparing stages 2 and 3, large effect sizes were seen in working memory (0.96, CI [0.08–1.80]) and cognitive ability (0.83 CI [0.01–1.63] indicating worsening scores with the advanced disease stage. The effect sizes difference between stage 1 and stage 2 and stage 2 and stage 3 were negligible across all neurocognitive outcomes.

**Table 5:** Cohens *d* analysis to compare neurocognitive scores by HIV disease staging.

	Language		Working memory		Inhibitory control		Cognitive ability		Set shifting		Cognitive flexibility	
	Effect size estimate	(95% CI)	Effect size estimate	(95% CI)	Effect size estimate	(95% CI)	Effect size estimate	(95% CI)	Effect size estimate	(95% CI)	Effect size estimate	(95% CI)
Stage 1 vs. 2	−0.35	−1.12 to 0.43	−0.10	−0.98 to 0.79	0.24	−0.62 to 1.09	−0.35	−1.11 to 0.42	0.26	−0.61 to 1.11	−0.50	−1.27 to 0.29
Stage 2 vs. 3	0.52	−0.28 to 1.32	**0** **.** **96**	**0.08–1.80**	0.50	−0.38 to 1.29	**0** **.** **83**	**0.01–1.63**	0.55	−0.30 to 1.39	−055	−1.37 to 0.29
Stage 1 vs. 3	0.10	−0.76 to 0.95	0.87	−0.18 to 1.89	0.73	−0.26 to 1.71	0.36	−0.51 to 1.22	0.69	−0.30 to 1.67	−0.80	−1.71 to 0.13

Bold values represent *p*-values <0.05.

## Discussion

This study examined the neurocognitive outcomes of children exposed to and living with HIV compared to a representative HIV-unexposed control group. The project also explored the relationship between child neurocognitive outcomes and child biomedical factors (nutritional status) and caregivers’ caregiver's psychosocial factors (common mental disorders and parenting behaviour) on the Kenyan coast.

Overall, the results showed similar neurocognitive outcomes among the three child groups, however, there were some subtle differences between groups including cognitive ability scores whereby the CHEU group had significantly higher scores than the CHUU and CHEI children. Similar results have been reported in previous studies conducted in Africa showing comparable neurodevelopment in children regardless of HIV status (59, 60). However, these findings conflict with our prior hypothesis and with previous reports in the literature which indicate that CHEI group have poorer neurocognitive outcomes compared to HIV-uninfected children (15, 61–65). There are various potential explanations for these results. First, the majority of the CHEU were school-going compared to the CHEI and CHUU group. The schooling status of CHEU could have explained why they performed better in these neurocognitive outcomes as previously documented by other studies (66). Second, the CHEI in this study were on ART which has been reported to reduce the incidence of HIV-related encephalopathy (which is linked to neurodevelopmental delays) by up to 50% (13). Third, this could be attributed to the benefits of current comprehensive HIV health-care management practices in Kenya (including PMTCT care services) whereby substantial attention has been given to CHEI and CHEU leaving out the CHUU, which could have affected the neurocognitive scores. Random selection of study participants and matching the cases to controls based on age and geographical location hopefully mitigated selection bias, but as CHEI and CHEU were recruited from the CCRC, this may have overestimated outcomes as children or families in follow up may perform better than those not on follow up. However, these results could also be a true reflection of neurocognitive outcomes of the three child groups based on the follow up CHEI and CHEU receive. Empirical evidence has shown that even when individuals have had early brain insults, under supportive conditions, they may learn and acquire compensatory skills that ensure they perform well in neuropsychological tests (24, 67).

The study found differences in cognitive scores between the CHEU compared to the CHEI and the CHUU. These results are comparable with previous findings from a prospective observational study on CHEU and uninfected community controls aged 24 months in Botswana (68). Arguably, the CHEU have frequent follow-ups as part of the PMTCT package which may provide avenues for implementation of additional services such as educational programs, which the community controls may not routinely access. Overall, these findings support the safety of in-utero exposure to maternal HIV and ART with respect to early cognitive development in CHEU. Of note, we did not measure gross motor and expressive language function which are the domains found to be affected in a recent meta-analysis of young CHEU (35). However, in this study the CHEU group scored lowest on receptive language, indicating the language domain may still be affected.

While comparing HIV stages 2 and 3, large effect sizes were seen in working memory and cognitive ability scores indicating worsening scores with the advanced disease stage. These results agree with previously reported findings whereby working memory and cognitive scores were related to the HIV disease stage (69, 70). For instance, Abubakar et al. (25) reported poorer psychomotor performance with advanced HIV disease staging in infants aged 6–35 months in Kilifi. This supports the continued need for early HIV treatment to halt the disease progression and slow down the negative impact of HIV on neurocognitive outcomes in children. Nonetheless, our findings demonstrate smaller effects in other neurocognitive outcomes compared with other research which showed evidence of an association between neurocognitive outcomes and HIV disease staging (71, 72). Notably, the results from the current study could be explained by the small sample size which could have lowered the power of the study to detect significant associations. Secondly, there could have been measurement bias in that the indicator used for disease staging may have been inadequate. In this study, disease staging was only measured using the WHO clinical staging which has been identified to have several limitations, including low sensitivity (73–75). The use of clinical disease staging with concurrent CD4 count and viral loads could have been better indicators of the HIV-disease stage and immunosuppression (76), but were not available in the current study.

There was a significant association between better nutritional status and increased cognitive ability scores. These results are consistent with previous findings from Kenyan infants which documented nutritional status as an independent predictor of various neurocognitive outcomes including language and psychomotor functioning (23–25). Other studies elsewhere have also reported the role of better nutrition on child neurodevelopmental outcomes (11, 70, 77–79). Our results, therefore, reiterate the substantial role of good nutrition in improving neurocognitive outcomes in children, suggesting the need to improve all children's nutritional status for optimal development.

There were no significant associations between parenting behaviour and neurocognitive outcomes. However, we noted a general trend of increased neurocognitive scores with improved parenting practices. Contrary to these findings, previous studies have documented the association between parenting behaviour and child neurodevelopment in children aged 6–12 years (80). Similarly, in a longitudinal study, parenting behaviour was significantly associated with executive function, implying that a supportive parenting environment can be a strong protective factor for a child's neurodevelopment amidst biological vulnerabilities (80). One possible explanation for the observed results is that young children are dependent on their caregivers for primary interactions which form the foundation for a healthy attachment and acquisition of developmental skills (81).

We observed a significant inverse association between CMDs and inhibitory control. We also observed a general reduction in scores across all neurocognitive scores with increased caregivers’ CMD scores, although not statistically significant. The lack of significant associations contrasts with previous findings from African studies which showed evidence of a relationship between caregiver's depression and child neurodevelopment (82–86). However, other studies have documented associations between maternal common mental disorders and poorer child neurocognition (87–90). The inconsistent evidence regarding the influence of caregivers’ mental health on child neurocognitive outcomes underscores the urgent need for further investigations in this topic. Currently, there is scarcity of research papers from LMICs in this area (87). Notably, in this study, caregivers of CHEI reported significantly higher CMDs scores and lower parenting behaviour scores compared to caregivers of CHUU and CHEU. The reportedly high CMDs scores in caregivers of CHEI and CHEU compared to CHUU have been described in the literature and could be partly attributed to the deterioration of their physical health, family breakdown, emotional distress and HIV-related stigma (91).

### Strengths and limitations of the study

This study is among the few quantitative studies examining the neurocognitive outcomes of HIV-infected, HIV-exposed uninfected and HIV-unexposed uninfected children within SSA. It is the first study of such kind in Kenya. The inclusion of two comparison groups in the analysis (CHEU and CHUU) was a particular strength of the study, enabling the comparison of neurocognitive outcomes across three child groups in the same setting.

Given the cross-sectional nature of this study, it was not possible to infer causality for the associations observed. Furthermore, the community control group was not actively screened for HIV. This implies that it is possible there were HIV-infected or exposed children among the controls, however, this is unlikely given the measures put in place to prevent this. We also note the possibility of residual bias despite adjusting for potential confounders, and that the CHUU group may have been disadvantaged given the lower schooling attendance which could have influenced group comparisons. Although child schooling status was adjusted for in the multivariable regression model, the number of years at school would have been a better measure of education, however these data were not available. Again, the CHEI and CHEU children were recruited from CCRC which may have overestimated outcomes as children not in follow up may perform worse than those on follow up. Additionally, data regarding the participants’ residential areas, specifically whether they were from rural or peri-urban settings, was not collected. Another major limitation of this project was a small sample size. This might have lowered the power to detect significant neurocognitive outcomes among the groups and associations. Additionally, the use of self-reported interviewer-administered measures of depression, SES and parenting behaviour to caregivers may have led to social desirability bias.

## Conclusion

This study demonstrated similar neurocognitive outcomes among the children living with HIV, those exposed to HIV but uninfected and the community controls, although some subtle differences were noted for cognitive abilities where the CHEU performed better than the other child groups. These results suggest that early use of ART and the comprehensive care services received by CHEI and CHEU may have mitigated potential neurodevelopmental deficits. The study further confirms the importance of child nutrition and caregiver mental health for optimising child neurodevelopment, underscoring the need for continued strategies to address these issues. Well-designed longitudinal studies are needed to further explore these findings.

## Data Availability

Data used in this article should be made through the coordinator of the data governance committee of the KEMRI Wellcome Trust Research Programme. The committee will review the application and advise as appropriate and ensure that uses are compatible with the consent obtained from participants for data collection. Requests to access the datasets should be directed to dgc@kemri-wellcome.org.
